# Association between initial body temperature on hospital arrival and neurological outcome among patients with out-of-hospital cardiac arrest: a multicenter cohort study (the CRITICAL study in Osaka, Japan)

**DOI:** 10.1186/s12873-022-00641-5

**Published:** 2022-05-14

**Authors:** Satoshi Yoshimura, Takeyuki Kiguchi, Taro Irisawa, Tomoki Yamada, Kazuhisa Yoshiya, Changhwi Park, Tetsuro Nishimura, Takuya Ishibe, Yoshiki Yagi, Masafumi Kishimoto, Sung-Ho Kim, Yasuyuki Hayashi, Taku Sogabe, Takaya Morooka, Haruko Sakamoto, Keitaro Suzuki, Fumiko Nakamura, Tasuku Matsuyama, Yohei Okada, Norihiro Nishioka, Satoshi Matsui, Shunsuke Kimata, Shunsuke Kawai, Yuto Makino, Tetsuhisa Kitamura, Taku Iwami, on behalf of the, CRITICAL Study Group Investigators

**Affiliations:** 1 Department of Preventive Services, Kyoto University School of Public Health, Kyoto, Japan; 2 Critical Care and Trauma Center, Osaka General Medical Center, Osaka, Japan; 3 Department of Traumatology and Acute Critical Medicine, Osaka University Graduate School of Medicine, Suita, Japan; 4 Emergency and Critical Care Medical Center, Osaka Police Hospital, Osaka, Japan; 5 Department of Emergency and Critical Care Medicine, Kansai Medical University, Takii Hospital, Moriguchi, Japan; 6 Department of Emergency Medicine, Tane General Hospital, Osaka, Japan; 7 Department of Critical Care Medicine, Osaka City University, Osaka, Japan; 8 Department of Emergency and Critical Care Medicine, Kindai University School of Medicine, Osaka-, Sayama, Japan; 9 Osaka Mishima Emergency Critical Care Center, Takatsuki, Japan; 10 Osaka Prefectural Nakakawachi Medical Center of Acute Medicine, Higashi-, Osaka, Japan; 11 Senshu Trauma and Critical Care Center, Osaka, Japan; 12 Senri Critical Care Medical Center, Saiseikai Senri Hospital, Suita, Japan; 13 Traumatology and Critical Care Medical Center, National Hospital Organization Osaka National Hospital, Osaka, Japan; 14 Emergency and Critical Care Medical Center, Osaka City General Hospital, Osaka, Japan; 15 Department of Pediatrics, Osaka Red Cross Hospital, Osaka, Japan; 16 Emergency and Critical Care Medical Center, Kishiwada Tokushukai Hospital, Osaka, Japan; 17 Department of Emergency and Critical Care Medicine, Kansai Medical University, Hirakata, Osaka, Japan; 18 Department of Emergency Medicine, Kyoto Prefectural University of Medicine, Kyoto, Japan; 19 Division of Environmental Medicine and Population Sciences, Department of Social and Environmental Medicine, Graduate School of Medicine, Osaka University, Osaka, Japan; 20 Department of Preventive Services, School of Public Health, Kyoto University, Kyoto, Japan, Postal code: 606-8501, YoshidaHonmachi, Sakyo, Kyoto, Japan

**Keywords:** Out-of-hospital cardiac arrest, Initial body temperature, Cardiopulmonary resuscitation, Neurological outcomes

## Abstract

**Background:**

The association between spontaneous initial body temperature on hospital arrival and neurological outcomes has not been sufficiently studied in patients after out-of-hospital cardiac arrest (OHCA).

**Methods:**

From the prospective database of the Comprehensive Registry of Intensive Care for OHCA Survival (CRITICAL) study in Osaka, Japan, we enrolled all patients with OHCA of medical origin aged > 18 years for whom resuscitation was attempted and who were transported to participating hospitals between 2012 and 2019. We excluded patients who were not witnessed by bystanders and treated by a doctor car or helicopter, which is a car/helicopter with a physician. The patients were categorized into three groups according to their temperature on hospital arrival: ≤35.9 °C, 36.0–36.9 °C (normothermia), and ≥ 37.0 °C. The primary outcome was 1-month survival, with a cerebral performance category of 1 or 2. Multivariable logistic regression analyses were performed to evaluate the association between temperature and outcomes (normothermia was used as the reference). We also assessed this association using cubic spline regression analysis.

**Results:**

Of the 18,379 patients in our database, 5014 witnessed adult OHCA patients of medical origin from 16 hospitals were included. When analyzing 3318 patients, OHCA patients with an initial body temperature of ≥37.0 °C upon hospital arrival were associated with decreased favorable neurological outcomes (6.6% [19/286] odds ratio, 0.51; 95% confidence interval, 0.27–0.95) compared to patients with normothermia (16.4% [180/1100]), whereas those with an initial body temperature of ≤35.9 °C were not associated with decreased favorable neurological outcomes (11.1% [214/1932]; odds ratio, 0.78; 95% confidence interval, 0.56–1.07). The cubic regression splines demonstrated that a higher body temperature on arrival was associated with decreased favorable neurological outcomes, and a lower body temperature was not associated with decreased favorable neurological outcomes.

**Conclusions:**

In adult patients with OHCA of medical origin, a higher body temperature on arrival was associated with decreased favorable neurologic outcomes.

## Introduction

The measurement of body temperature (BT) on admission is important, as it is a vital sign that reflects the patient’s condition at the onset of out-of-hospital cardiac arrest (OHCA), which can be easily performed in the emergency room and is recommended by the guidelines [1]. The association between BT measured after hospital arrival and outcomes has been shown in several studies [2, 3], but the association between high BT on hospital arrival and outcomes has not been sufficiently evaluated. Additionally, some studies have reported that hypothermia at hospital arrival is associated with poor outcomes [4–7]. However, important confounding factors, such as the effect of transport time, were not adjusted in these studies, and further efforts to adjust for potential confounders are needed to show an association between BT and neurological outcomes after OHCA.

To improve the survival rate after OHCA by providing evidence-based therapeutic strategies and medical systems, we launched the Comprehensive Registry of Intensive Care for OHCA Survival (CRITICAL) study, which is a multicenter, prospective observational data registry in Osaka, Japan, designed to accumulate both pre-and in-hospital data on OHCA treatment [8]. Using this database, we evaluated the association between initial BT on hospital arrival and favorable neurological outcomes among adult patients with OHCA.

## Methods

### Study design and setting

In this study, we analyzed the database of the CRITICAL study, which is a multicenter prospective observational data registry designed to accumulate both pre-and in-hospital information on OHCA treatment. A complete description of the study methodology has been previously reported [8].

### Population and settings

The target area of the CRITICAL study is Osaka Prefecture in Japan, which has an area of 1897 km^2^ and a residential population of 8,839,469 inhabitants as of 2015; 48.1% of the population are males, 25.8% of whom are ≥65 years old [9]. Osaka had 535 hospitals (108,569 beds) in 2013 [10]. A total of 280 hospitals accepted emergency patients from ambulances. Of these, 16 hospitals have critical care medical centers (CCMCs) that can accept severely ill emergency patients [10]. Fifteen CCMCs and one non-CCMC with an emergency care department in Osaka participated in this study. Approximately 30% of patients with OHCA in Osaka are transported to and treated at CCMCs [10]. This CRITICAL study, including a retrospective analysis, was approved by the ethics committee of the Kyoto University (R-1045). The requirement for informed consent was waived.

### Data collection and quality control

This registry’s data collection and quality control details have been reported elsewhere [8]. Pre-hospital data on OHCA patients were obtained from the All-Japan Utstein Registry, and data were uniformly collected according to the Utstein-style international guidelines for reporting OHCAs. Each EMS personnel completed a data form in cooperation with the attending physicians in charge of the patient. In this study, a doctor car/helicopter was defined as an ambulance/helicopter in which a physician traveled from the scene of a patient’s collapse to the patient’s arrival at the hospital. The unified protocol on how to dispatch the physician to the location of the occurrence of cardiac arrest and the role of the pre-hospital physician in Japan are not clearly defined. For in-hospital data collection and quality control, the CRITICAL registry has collected substantial data on patients with OHCA after arrival at the hospital, as detailed have been provided in a previous paper. For the current registry, anonymized data were entered into the web sheet either by the physician or medical staff in collaboration with the attending physician in charge of the patient. The pre-hospital and in-hospital data were uploaded to the registry system, logically checked by the computer system, and confirmed by the working group, which consisted of experts in emergency medicine and clinical epidemiology.

### Study patients

We enrolled all consecutive patients with OHCA (aged ≥18 years) for whom resuscitation was attempted and then transported to the participating institutions between January 1, 2012, and December 31, 2019. This study excluded patients with OHCA who did not receive cardiopulmonary resuscitation (CPR) from physicians after hospital arrival and those who disagreed with our registry (refusal by the patient or the patient’s family). Additionally, patients with OHCA who were not of medical origin, without witness, transported by a doctor car or helicopter, and whose BT on arrival was not available were excluded. The requirement for obtaining individual informed consent to review patient outcomes was waived.

### Outcomes

The primary outcome measure was 1-month survival with favorable neurological outcomes after OHCA. A favorable neurological outcome was defined as a cerebral performance category (CPC) score of 1 or 2 [11]. The secondary outcome measure was the 1-month survival. Outcome data were also prospectively collected and included as follows [8]: 1-month survival and neurological status 1-month after OHCA occurrence, using the CPC scale (category 1, normal cerebral performance; category 2, moderate cerebral disability; category 3, severe cerebral disability; category 4, coma or vegetative state; and category 5, death/brain death). The survivors underwent neurologic assessment by the physician in charge 1-month after the event. The patients in this analysis included those with valid BT data available on hospital arrival. Temperatures were recorded from the ear, rectum, urinary bladder, axilla, and others. The patients were categorized into three groups according to their temperature on arrival at the hospital, based on clinical significance according to previous studies [7, 12]. The low BT group had an initial temperature of 35.9 °C or below; the normothermia group was defined as the group with a temperature from 36.0 °C to 36.9 °C, and the other group was the higher temperature group defined as the temperature of ≥37.0 °C.

### Statistical analysis

We described the characteristics of the patients in each BT group. Data are presented as mean ± standard deviation for continuous variables and percentages for categorical variables. Categorical data were compared using the chi-square test. Continuous data were compared using the Kruskal–Wallis test. To assess the association between BT and outcomes, we used a univariable logistic regression model for crude odds ratios (ORs) and performed a multivariable logistic regression analysis to adjust for potential resuscitation factors associated with 1-month survival and 1-month survival with favorable neurological outcomes, and the ORs and 95% confidence intervals (CIs) were calculated. The independent variables considered in this analysis included the following: age (continuous value), sex (male, female), origin of arrest (cardiac, non-cardiac), bystander CPR (no, yes), initial rhythm (shockable [ventricular fibrillation and pulseless ventricular tachycardia] or non-shockable [pulseless electrical activity and asystole], which were defined as the first documented rhythm at the scene), time from emergency medical service (EMS) call to the hospital (continuous variable), return of spontaneous circulation before hospital admission (no, yes), season (spring: March, April, and May; summer; June, July, and August; autumn: September, October, and November; and winter: December, January, and February). This study excluded unwitnessed cases from the analysis. First, because the Utstein template recommends focusing on witnessed cases for resuscitation [11], and second, it is difficult to assess the recorded temperature of unwitnessed cases together with witnessed cases because the time of collapse is not known among unwitnessed cases. For the second analysis, restricted cubic splines (RCS) were used to detect the possible nonlinear dependency of the relationship between BT and outcomes using four knots at prespecified locations according to the percentiles of the distribution of BT (5th, 25th, 75th, and 95% percentiles) [13]. In the analysis, we conducted a post-hoc analysis of the linearity of the association between BT and outcome among OHCA patients, and we found a statistically nonlinear association between neurological outcome and BT among them. Furthermore, we compared the fitness of the linear and nonlinear models for the subjects. As a result, the nonlinear model was better fitted than the linear model by the F test. Moreover, we described the distribution of BT by the first documented rhythm on EMS arrival (ventricular tachycardia [VF]/pulseless ventricular tachycardia [pVT], pulseless electrical activity [PEA]/asystole) and performed subgroup analyses by rhythm using multivariate logistic regression analysis. All *p*-values were two-sided, and statistical significance was set at *p* < 0.05. All statistical analyses were performed using STATA version 16.0 SE software (Stata Corp LP) and R studio (Version 1.2.5033). The aforementioned dose-response analyses by RCS were performed using R studio with the package of “rms.”

## Results

### Study participants

**Fig. 1:**
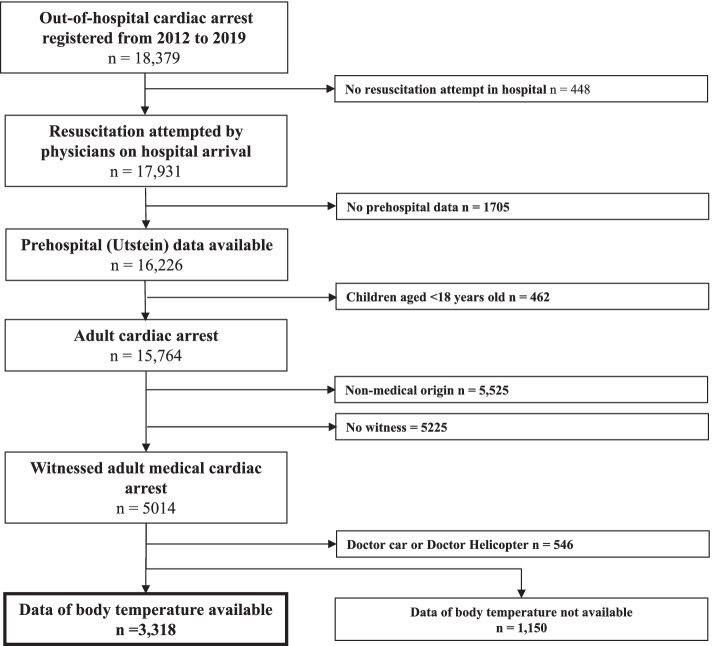
Patient flow

Figure 1 shows an overview of the study population. From 18,379 patients with OHCA between 2012 and 2019, 3318 were included in the final analysis (*n* = 15,061 excluded) after excluding patients not resuscitated, without pre-hospital data, pediatric patients, non-medical origin (external causes including traffic injury, fall, hanging, drowning, asphyxia, drug overdose, or any other external cause), and without witnesses. Table 1 shows the baseline characteristics of the 3318 patients with OHCA. We also showed the characteristics of 1150 patients whose BT data were missing. There were 1358 patients with low BT (≤35.9 °C), 695 with normothermia (36.0–36.9 °C), and 195 with high BT (≥37.0 °C). Of these, the proportion of patients with VF/pVT as the first documented rhythm at EMS arrival were 24.9% in the low BT group, 25.8% in the normothermia group, and 12.9% in the high BT group, respectively. The proportion of PEA/asystole was highest in the high BT group (77.6%). The prevalence of cardiac origin did not differ among the three groups (85.4, 85.3, and 84.3%, respectively). The average time from call to the hospital was 35.1 min, 36.8 min, and 34.6 min. During this season, the proportion of summer was lowest in the low BT group (16.3%). The low BT group had a higher proportion of measurement sites in the ear (29.6%) than the other groups.

**Table 1:** Patient Characteristic

Body temperature on arrival to hospital, °C	Overall		Missing		≦35.9		36.0–36.9		37.0≦		
	(***n*** = 4468)		(***n*** = 1150)		(***n*** = 1932)		(***n*** = 1100)		(***n*** = 286)		***P*** value*
Age, years, mean (SD)	70.28	(15.0)	71.81	(14.6)	70.1	(14.8)	69.1	(15.2)	69.8	(16.1)	0.001
Men, n (%)	2996	(67.1)	741	(64.4)	1264	(65.4)	781	(71.0)	210	(73.4)	0.001
First documented rhythm at EMS arrival, n (%)											< 0.001
VF/pulseless VT	1072	(24.0)	269	(23.4)	482	(24.9)	284	(25.8)	37	(12.9)	
PEA/Asystole	3075	(68.8)	816	(71.0)	1304	(67.5)	733	(66.6)	222	(77.6)	
Others	321	(7.2)	65	(5.7)	146	(7.6)	83	(7.5)	27	(9.4)	
Bystander-initiated CPR, n (%)	1760	(39.4)	424	(36.9)	794	(41.1)	453	(41.2)	89	(31.1)	0.004
Bystander-initiated AED, n (%)	153	(3.4)	27	(2.3)	71	(3.7)	47	(4.3)	8	(2.8)	0.462
Origin											
Cardiac, n (%)	3843	(86.0)	1015	(88.3)	1649	(85.4)	938	(85.3)	241	(84.3)	0.889
Non-Cardiac											0.006
Cerebrovascular disease, n (%)	187	(4.2)	26	(2.3)	80	(5.9)	69	(9.9)	12	(6.2)	
Respiratory disease, n (%)	283	(6.3)	51	(4.4)	141	(10.4)	61	(8.8)	30	(15.4)	
Malignancy, n (%)	148	(3.3)	54	(4.7)	49	(3.6)	30	(4.3)	15	(7.7)	
Time from call to hospital, min (mean (SD))	35.8	(16. 5)	36.2	(16. 5)	35.1	(15.3)	36.8	(18.5)	34.6	(15.8)	0.018
Season, n (%)											< 0.001
Spring	1070	(23.9)	250	(21.7)	494	(25.6)	265	(24.1)	61	(21.3)	
Summer	916	(20.5)	233	(20.3)	315	(16.3)	292	(26.5)	76	(26.6)	
Autumn	1117	(25.0)	299	(26.0)	469	(24.3)	285	(25.9)	64	(22.4)	
Winter	1365	(30.6)	368	(32.0)	654	(33.9)	258	(23.5)	85	(29.7)	
BT measeurement site n, (%)											< 0.001
Ear	674	(20.6)	N/A		564	(29.6)	78	(7.2)	32	(11.3)	
Rectal	71	(2.2)	N/A		28	(1.5)	22	(2.0)	21	(7.4)	
Urinary bladder	186	(5.7)	N/A		117	(6.1)	53	(4.9)	16	(5.7)	
Axillary	2035	(62.3)	N/A		1083	(56.8)	747	(69.2)	205	(72.7)	
Others	302	(9.2)	N/A		115	(6.0)	179	(16.6)	8	(2.8)	

*P value is caluculated for 3 temperature categories. *EMS* Emergency medical service, *CPR* Cardiopulmonary resuscitation, *AED* Automated external defibrillator, *VF* Ventricular fibrillation, *VT* Ventricular tachycardia, *PEA* Pulseless electrical activityr, *SD* Standard deviation, *BT* Body temperature, *N/A* Not applicable

The in-hospital advanced treatment and drug administration among patients with OHCA are shown in Table 2. After arrival in the hospital, for each group (low BT, normothermia, and high BT groups), 17.3, 13.9, and 11.5% of the patients received defibrillation; 12.8, 7.6, and 5.6% extracorporeal life support; 15.5, 17.3, and 10.1% target temperature management; and 10.1, 10.4, and 6.3% received 34 °C management, respectively.

**Table 2:** In-hospital management and prognosis

Body temperature on arrival, °C	Overall		Missing		≦35.9		36.0–36.9		37.0≦		
	(***n =*** 4468)		(***n =*** 1150)		(***n =*** 1932)		(***n =*** 1100)		(***n =*** 286)		***P*** value*
Defibrillation, n (%)	758	(17.0)	237	(20.6)	335	(17.3)	153	(13.9)	33	(11.5)	0.006
Tracheal intubation after hospital arrival, n (%)	2811	(62.9)	701	(61.0)	1224	(63.4)	710	(64.5)	176	(61.5)	0.607
Extracorporeal life support, n (%)	470	(10.5)	122	(10.6)	248	(12.8)	84	(7.6)	16	(5.6)	< 0.001
Intra-aortic balloon pumping, n (%)	399	(8.9)	89	(7.7)	213	(11.0)	85	(7.7)	12	(4.2)	< 0.001
Coronary angiography, n (%)	878	(19.7)	164	(14.3)	445	(23.0)	233	(21.2)	36	(12.6)	< 0.001
Percutaneous coronary intervention, n (%)	433	(9.7)	87	(7.6)	226	(11.7)	105	(9.5)	15	(5.2)	0.002
Success of reperfusion, n (%)	362	(90.7)	72	(90.0)	186	(89.9)	93	(93.9)	11	(84.6)	0.368
Target temperature management, n (%)	600	(13.4)	81	(7.0)	300	(15.5)	190	(17.3)	29	(10.1)	0.012
34 °C management, n (%)	382	(8.5)	54	(4.7)	196	(10.1)	114	(10.4)	18	(6.3)	0.268
Time from arrival to achivement of Targeted Temperature, min (SD)	373	(364)	416	(331)	377	(418)	353	(294)	343	(264)	0.745
Time from initiation to achivement of Targeted Temperature, min (SD)	238	(367)	295	(552)	240	(380)	215	(243)	224	(259)	0.726
SD, standard deviation. *P value is caluculated for 3 temperature categories.											

### Outcomes

**Fig. 2:**
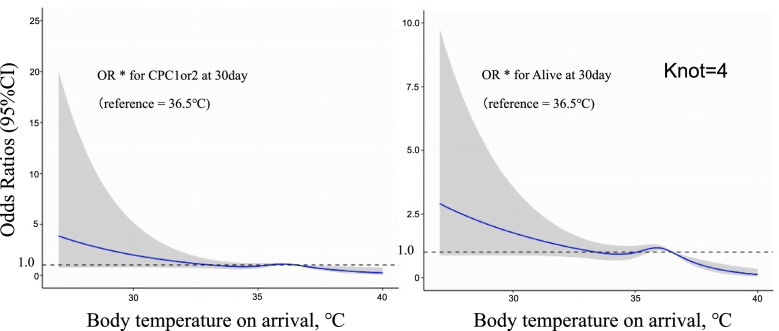
The association between BT and outcomes. Restricted cubic spline curve of temperature on arrival and adjusted risk of outcomes: (left) BT on arrival and OR for CPC 1 or 2 30-day after OHCA and (right) BT on arrival and OR for survival 30-day after OHCA. Curves represent adjusted OR (solid line) and 95% confidence intervals (shades). *ORs were adjusted for age, sex, origin of arrest, bystander cardiopulmonary resuscitation, initial rhythm, time from EMS call to the hospital, ROSC before hospital, and season. EMS, emergency medical services; VF, ventricular fibrillation; VT, ventricular tachycardia; PEA, pulseless electrical activity; CPC, cerebral performance category; BT, body temperature; OR, odds ratio; CI, confidence interval; ROSC, return of spontaneous circulation

Table 3 presents the results of the primary analyses. Among adult patients, low and high BT on hospital arrival were related to the decreased favorable neurological outcome in univariable analysis; crude ORs of 0.64 (95% CI, 0.51–0.79) and 0.36 (95% CI, 0.22–0.59) for the low BT and high BT groups, respectively, compared to the normothermia group (reference), and in multivariable analysis after adjusting for potential confounders; adjusted ORs of 0.78 (95% CI, 0.56–1.07) and 0.51 (95% CI, 0.27–0.95) for low BT and high BT, respectively, compared to a BT of 36.0–36.9 °C (reference). For the secondary outcome, the high temperature was associated with a decreased 1-month survival in both univariable and multivariable analysis; crude OR of 0.39 (95% CI, 0.26–0.59) and adjusted OR of 0.44 (0.26–0.72). Figure 2 shows the association between BT on hospital arrival and the outcomes in the secondary analysis. We modeled the relationship between BT on arrival and the outcomes using RCS models with four knots. There were significant nonlinear dose-response associations between high BT on arrival and outcomes (Fig. 2).

**Table 3:** OR for 30-day CPC 1or 2 and 30-day survival after OHCA

	Overall		Missing		(≦35.9)°C		(36.0–36.9)°C		(≧37.0)°C	
All rhythm										
CPC 1 or 2 one month after OHCA, n (%)	473	(10.6)	60	(5.2)	214	(11.1)	180	(16.4)	19	(6.6)
Crude OR (95% CI)	N/A		N/A		0.64	(0.51–0.79)	Reference		0.36	(0.22–0.59)
Adjusted OR* (95% CI)	N/A		N/A		0.78	(0.56–1.07)	Reference		0.51	(0.27–0.95)
One-month survival, n (%)	732	(16.4)	103	(9.0)	353	(18.3)	247	(22.5)	29	(10.1)
Crude OR (95% CI)	N/A		N/A		0.77	(0.64–0.93)	Reference		0.39	(0.26–0.59)
Adjusted OR* (95% CI)	N/A		N/A		0.96	(0.74–1.24)	Reference		0.44	(0.26–0.72)

*ORs were adjusted for age, sex, origin of arrest, bystander cardiopulmonary resuscitation, initial rhythm, time from EMS call to the hospital, ROSC before hospital and season; *VF* Ventricular fibrillation, *VT* Ventricular tachycardiaM, *PEA* Pulseless electrical activityM *CPC* Cerebral performance category, *OR* Odds ratio, *CI* Confidence interval, *ROSC* Return of spontaneous circulation, *N/A* Not applicable

**Fig. 3:**
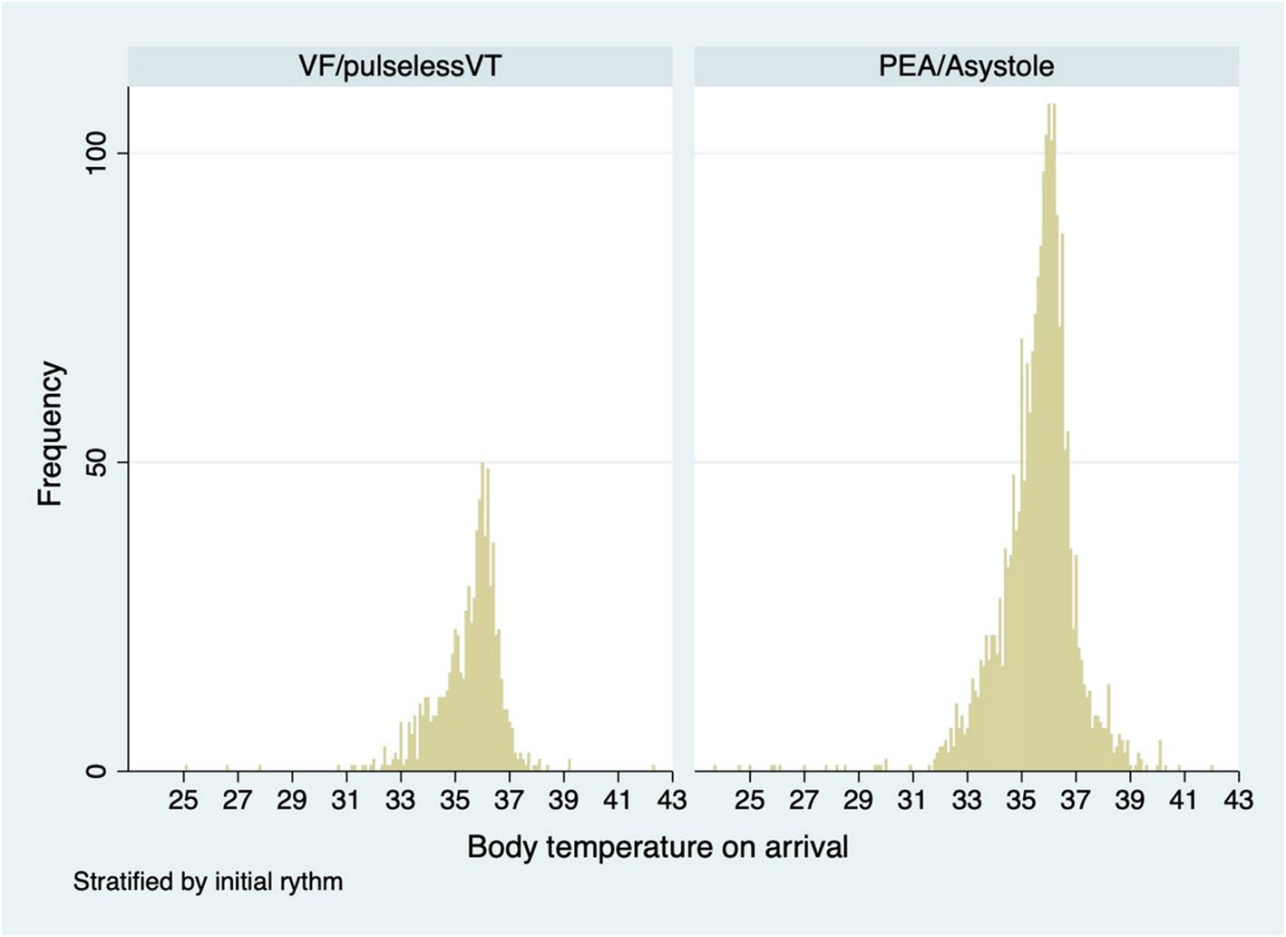
Distribution of BT on hospital arrival. BT, body temperature

For the subgroup analysis, we depicted the distribution of BT in both the shockable (VF/pVT) and non-shockable (PEA/asystole) groups (Fig. 3). BT was more broadly distributed towards high BT in the non-shockable group. Table 4 shows the results of the subgroup analysis. Low and high BT on arrival were not related to decreased favorable neurological outcomes in either univariate or multivariate analyses in the shockable group. The high temperature was associated with decreased favorable neurological outcomes in multivariable analysis among the non-shockable group, with an adjusted OR of 0.29 (0.10–0.86).

**Table 4:** Subgroup analysis: OR for 30-day CPC 1or2 after OHCA

	Overall		Missing		(≦35.9)°C		(36.0–36.9)°C		(≧37.0)°C	
Shockable, VF/pulseless VT										
CPC 1 or 2 1 month after OHCA, n (%)	319	(29.8)	47	(17.5)	139	(28.8)	124	(43.7)	9	(24.3)
Crude OR (95% CI)	N/A		N/A		0.52	(0.38–0.71)	Reference		0.41	(0.19–0.91)
Adjusted OR* (95% CI)	N/A		N/A		0.69	(0.45–1.07)	Reference		0.68	(0.25–1.84)
Non-shockable, PEA/asystole										
CPC 1 or 2 1 month after OHCA, n (%)	87	(2.8)	9	(1.1)	45	(3.5)	28	(3.82)	5	(2.3)
Crude OR (95% CI)	N/A		N/A		0.90	(0.56–1.46)	Reference		0.58	(0.22–1.52)
Adjusted OR* (95% CI)	N/A		N/A		0.76	(0.44–1.32)	Reference		0.29	(0.10–0.86)

*ORs were adjusted for age, sex, origin of arrest, bystander cardiopulmonary resuscitation, time from EMS call to the hospital, ROSC before hospital and seasoN, *ROSC* Return of spontaneous circulation, *OR* Odds raio, *CPC* Cerebral Performance Category, *OHCA* Out-of-hospital cardiac arrest, *EMS* Emergency medical service, *VF* Ventricular fibrillationM, *VT* Ventricular tachycardia, *PEA* Pulseless electrical activity, *CPC* Cerebral performance category, *OR* Odds ratio, *CI* Confidence interval, *N/A* Not applicable

## Discussion

### Summary

Using data from a multicenter, prospective observational registry of OHCA in Osaka, Japan, we demonstrated an association between high BT on hospital arrival and decreased favorable neurological outcomes among adult patients with OHCA. One-month survival with favorable neurological outcomes after OHCA was lower in the high-BT group than in the normothermia group. Low BT on hospital arrival was not associated with decreased favorable outcomes in the primary analysis; additionally, there was no dose-response relationship between them in the RCS analysis.

### Comparison with previous studies

Previous studies have reported that hyperthermia within 1–24 or 48 h of presentation is associated with decreased favorable neurological outcomes [2, 3]. In this study, hyperthermia on hospital arrival was associated with decreased favorable outcomes after adjusting for confounders, and secondary analysis showed an exposure-response relationship between high BT and outcomes.

In the subgroup analysis, among patients with OHCA with non-shockable rhythm, the high BT group had significantly fewer favorable neurological outcomes than the normothermia group, while it was not statistically significant among patients with shockable rhythm. The high BT group in this study had fewer cases of VF/pVT and more cases of PEA/systole than the low BT and normothermia groups. The non-shockable group has more non-cardiogenic diseases and poorer outcomes [14–16]. Cardiac arrest due to sepsis had a poor outcome in a previous study [17], and causative febrile diseases of cardiac arrest, such as sepsis, may have affected the poor outcome in the high BT group. In this study, the high BT group had lower rates of CPR and higher rates of respiratory disease of non-cardiac origin, and AEDs were less often used. Furthermore, they were less often defibrillated and underwent less extracorporeal life support, IABP, CAG, and PCI. These findings may indicate that worse circumstances at arrest, perhaps different comorbidity burdens, and a different patient profile in the high BT group might have affected the outcomes.

In this study, hypothermia was not associated with decreased favorable outcomes in the main analysis and did not show an exposure-response relationship in the secondary analysis. Previous studies have reported that patients with OHCA with hypothermia have a poor outcome [4–7], and some of these patients may have been in a hypocirculatory state or in shock for a long time. However, it is well known that patients with accidental hypothermia have a relatively good outcome [18, 19]. The low BT group would be diverse, and the background in the pre-hospital setting and the causative disease of patients with OHCA with initial hypothermia may have affected the results.

### Interpretation of the results and possible implications

The results of this study, in which hyperthermia on hospital arrival was associated with the outcome of patients with endogenous OHCA, provide further implications for improving clinical outcomes in OHCA patients. Although the cause of hyperthermia in OHCA patients presenting with hyperthermia was unknown in this study, future studies to determine their causes in the hyperthermia group, as well as the presence of complications such as sepsis [16], which is considered to have a poor prognosis, might lead to improved outcomes in these patients. In addition, it may be reasonable to measure the initial body temperature of patients with OHCA upon arrival at the hospital to identify the pathogenesis of hyperthermia (such as sepsis) and aggressively treat the underlying disease. Further studies are needed to verify the treatment and pathogenesis of hyperthermia in patients with OHCA of medical origin.

### Limitations

This study has several limitations. First, it was conducted in emergency centers in urban or suburban areas in Japan, and there is a possibility of selection bias. In addition, the cause of hypothermia in this region may differ from that in other studies because it is not a cold region. Second, the temperature measurement sites varied between the ear, rectum, urinary bladder, and axillary region, with the ear being more common in hypothermic patients. Third, the current study results could be interpreted with caution, as the superficial temperature, such as axillary temperature, may be lower than the deep-body temperature. “However, the BT of the patients who potentially have unfavorable outcomes at the scene would be usually observed by the superficial temperature.” In addition, patients whose BT was not measured were excluded from the analysis, which may have affected the results. Patients without BT measurements may be more likely to have a poor outcome or withdraw resuscitation early. Fourth, the location (home or outside) of the OHCA was not collected in this registry, which might have led to bias in the results. Lastly, we believe that these did not affect the relationship between hyperthermia and outcome but may have resulted in poorer outcomes in hypothermic patients.

## Conclusions

In patients with OHCA of medical origin, high BT on hospital arrival was associated with decreased favorable neurological outcomes compared to normothermia among patients with OHCA with non-shockable rhythm, but not in patients with a shockable rhythm.

## Data Availability

The dataset supporting the conclusions of this study is available from the corresponding author upon reasonable request.

## Abbreviations

AEDs: Dissemination of public-access automated external defibrillators
BT: Body temperature
CCMC: Critical care medical center
CI: Confidence interval
CPC: Cerebral performance category
CRITICAL: Comprehensive Registry of Intensive Care for OHCA Survival
CPR: Cardiopulmonary resuscitation
EMS: Emergency medical services
FDMA: Fire and Disaster Management Agency
OHCA: Out-of-hospital cardiac arrest
OR: Odds ratio
PEA: Pulseless electrical activity
pVT: Pulseless ventricular tachycardia
RCS: Restricted cubic splines
ROSC: Return of spontaneous circulation
TTM: Target temperature management
VF: Ventricular fibrillation
VT: Ventricular tachycardia
